# Playing with Expectations: A Contextual View of Humor Development

**DOI:** 10.3389/fpsyg.2016.01392

**Published:** 2016-09-20

**Authors:** Gabriella Airenti

**Affiliations:** Center for Cognitive Science, Department of Psychology, University of TorinoTorino, Italy

**Keywords:** humor development, communicative games, teasing, irony, theory of mind

## Abstract

In the developmental literature, the idea has been proposed that young children do not understand the specificity of non-literal communicative acts. In this article, I focus on young children’s ability to produce and understand different forms of humor. I explore the acquisition of the communicative contexts that enable children to engage in humorous interactions before they possess the capacity to analyze them in the terms afforded by a full-fledged theory of mind. I suggest that different forms of humor share several basic features and that we can construct a continuum from simple to sophisticated forms. In particular, I focus on teasing, a form of humor already present in preverbal infants that is also considered a typical feature of irony. I argue that all forms of humor can be regarded as a type of interaction that I propose to call “playing with expectations.”

## Introduction

In studies of communication, the interpretation of non-literal, indirect or figurative meaning occupies a distinct place. Various views have been advanced on this topic. In pragmatics, the classical two-stage theories distinguish a primary literal interpretation from a non-literal secondary interpretation, which can be developed only through the analysis and failure of the former (Grice, 1957, 1969; Searle, 1969). Subsequent psycholinguistic studies show the cognitive implausibility of the classical perspective (see, for instance, Clark and Lucy, 1975), and more recent theories analyze the multifaceted aspects of non-literal meaning interpretation (Wilson and Sperber, 1992; Giora, 1997). It is generally agreed that the differences between models correspond to the way context is analyzed and considered (Gibbs and Colston, 2012).

Non-literal communication includes numerous forms, such as indirect speech acts, metaphors, jokes, irony, and hyperbole. These forms occur commonly in everyday adult communication. Do they also occur in children’s communication? Certainly, when communicating with children, adults do not refrain from using non-literal expressions. Consider, for instance, the following examples:

- *Wanna go for a bike ride?*
- *You have a full dish in front of you.*
- *Your brother is an ogre.*
- *Tom’s cat is as big as an elephant.*
- *I love children who keep their rooms clean.*

When are children able to master these forms (i.e., to comprehend and produce them)? Are all of them cognitively equivalent? The few systematic studies that have been conducted suggest that acquisition does not follow a unique progression. Some forms are simpler to master than others (Bosco et al., 2013). It is therefore important to understand the reasons for differences in the ease of comprehension and to delineate specific paths of acquisition.

One hypothesis asserts that different forms of non-literal communication can be distinguished based on the role of theory of mind (ToM) abilities. The most demanding tasks require developed ToM abilities to comprehend the speaker’s meaning. For instance, according to Winner (1988), children comprehend metaphors before irony because understanding metaphors does not involve questioning the speaker’s beliefs, whereas comprehending irony involves attributing second-order beliefs to the speaker. In this paper, I argue that children may perform complex non-literal communicative acts before developing full-fledged ToM abilities.

An important question arises concerning the relationship between use and interpretation. Adults and children differ markedly with respect to this relationship. Theories may differ on how the chain of inferences that enables interpretation is constructed. Nonetheless, adults are undoubtedly able to interpret non-literal communication. If an adult laughs at a joke, we presume that s/he has understood its humor, and if s/he produces a joke, we assume that s/he has intentionally produced humor. When children produce humor, however, we are unsure whether they do so intentionally. Does the fact that a child laughs at a joke indicate that s/he has understood it? If the child makes us laugh, did the child do so intentionally, or was the humor unintended such that we, the audience, are creating it? In the case of adults, we do not pose the problem of the meaning of comprehension. Instead, we presume that use and comprehension are linked. In the case of children, this link remains unclear.

In the developmental literature, the idea is often advanced that young children do not understand the specificity of non-literal communicative acts and cannot distinguish, for instance, between an ironic statement or a hyperbole and a lie (Peterson et al., 1983; Demorest et al., 1984; Winner and Leekam, 1991; Sullivan et al., 1995; Winner et al., 1998). For young children utterances are either true or false, and when they are false, they can only be lies. Thus, it is reasoned, young children cannot properly appreciate non-literal communication.

This perspective is limited; it highlights only the tasks at which young children fail. Conversely, I aim to understand what young children *are* able to do. I believe this perspective might help to reconstruct the developmental path and thus to more effectively understand mature comprehension of non-literal communication. In this article, I focus specifically on young children’s ability to produce and understand different forms of humor.

My argument proceeds as follows. I identify the forms of humor that children typically use through several examples drawn primarily from parents’ reports. I then discuss the difficulties highlighted in the literature regarding the definition and categorization of different forms of humor. I specifically address the relationship between humor and irony. I explore the acquisition of the communicative contexts that constitute the background that enables children to engage in humorous interactions before being able to analyze them using full-fledged ToM abilities. I assume that young children react differently to lies and to non-literal communication. Finally, I present a theoretical proposal: I argue that different forms of humor share some basic features and that we can construct a continuum from simple to sophisticated forms. I focus on teasing, a form of humor already present in preverbal infants that is also considered a typical feature of irony. I conclude that all forms of humor can be considered a type of interaction that I propose to call “playing with expectations.”

## Children’s Use of Humor^1^

Children are involved in humorous^1^ communicative interactions from a very young age (Groch, 1974; Bainum et al., 1984; Dubois et al., 1984; Bergen, 1989; Reddy, 1991, 2008; Loizou, 2005; Cameron et al., 2008; Hoicka and Akhtar, 2012; Mireault et al., 2012). From a developmental perspective, the earliest cases of humorous interactions are amusing situations that occur between infants and adults. Two cases are typical. Adults propose an amusing action, such as tickling, odd faces or sounds, or blowing a raspberry. Children playfully respond to the action, and the interaction becomes a shared game. Sometimes the child initiates the interaction, often inadvertently, with a gesture or a sound that provokes amusement in the adult. This amused response pleases the child, who intentionally repeats the gesture to obtain the same reaction, and the game becomes shared. These humorous games are non-verbal and simple. Reddy (2008) classifies them as *clowning*, or the violation of normal patterns of behavior to elicit amusement.

The other type of humor commonly observed with young children is *teasing*.

Consider two examples.

When asked to make the sound of a horse (Come fa il cavallo?), a 2.5-year-old girl answers, “Moo” (Muh) and laughs.

Another parent reports an incident with her daughter, also 2.5 years old: “I asked Becky, ‘What is the cat’s call?’ (Come fa il gatto?). She answered ‘chirp’ (cip cip) and laughed. Then, she corrected herself: ‘No, mom, it meows! (Ma no, mamma, fa miao miao).”

Reddy (2008) showed that this form of humor is precocious, starting at approximately 9 months of age. Relying on parents’ reports, Reddy distinguished three types of teasing in young children: *provocative non-compliance*, *offer and withdrawal* of an object or of the self and *disrupting others’ activities*. In all of these types, children playfully disturb an interaction by performing “the mis-expected” (Reddy and Mireault, 2015). As these authors note, teasing, even in its simplest forms, requires the display of cognitive abilities. In particular, the child must have expectations regarding the interlocutor’s actions. For instance, in an offer/withdrawal, the infant must expect the interlocutor to extend her arm, open her hand and wait for the child to release the object. The child also expects the interlocutor to express surprise and disappointment after the withdrawal, and this response is the source of amusement. The authors assert that the wide spectrum of typical cases of teasing observed in young children indicates that “the range of things infants can do to tease their parents seems as large as the expectations parents have of the infants” (*ibid*.).

More precisely, based on my analysis of the existing literature and the parents’ reports I collected, it appears that parents’ expectations exploited by young children may be either r*elational* or *linked to newly acquired skills*. As examples of the first situation, consider the cases of contradicting expectations of kissing or hugging, withdrawing at the last moment, or playing with parents’ fears of approaching a dangerous or precious (and forbidden) object and withdrawing at the last moment.

One example was observed in a 2.3-year-old girl. “The aunt asked her, ‘Marta, will you give me a kiss?,’ to which she replied: ‘No, never!’ (No, mai!). The aunt looked sad, and [the girl] smothered her with kisses.”

A good example of fears is reported in Corsaro (1997). Corsaro’s daughter had just begun climbing chairs and other objects that parents consider dangerous to climb. Once, she climbed onto the seatback of a large armchair. When her father attempted to remove her from the seatback, she smiled broadly. According to the author, she seemed to be saying, “Look, dad, where I have gone this time!”

Common examples of playing with skills include those introduced earlier, such as deliberately attributing the wrong calls to animals, calling the father “mom” or the mother “dad,” or claiming that the sister (or the grandmother or the aunt) is a male, whereas the brother (or the grandfather or the uncle) is a female. Children typically play with newly acquired skills, a tendency confirmed in the literature. Garvey (1977) includes the cases of misnaming in the form of social playing, which consists of playing with speech acts and discourse conventions. She suggests that as soon as children have learned a rule, they have fun distorting or exaggerating it. Dunn (1988) argues that such episodes, which characterize the beginning of the development of a sense of humor in children, are motivated by the pleasure of performing forbidden acts. The fact that young children perform this game with newly acquired abilities indicates intentional teasing. Children play with parents’ uncertainty, as parents may be unsure whether the child is making a joke or a genuine mistake. The expression of uncertainty or trouble is, in turn, the source of amusement.

What form of communication does this type of humor represent? In at least one of the examples mentioned, the child explicitly indicated that her first response was not a mistake but an intentional joke. Often, however, the child makes no explicit declaration but displays what parents identify as a pert or ironic smile.

If we consider the development of teasing in older children, we may add a new category to the categories identified by Reddy, namely, *mocking* (Airenti and Angeleri, submitted).

Consider an example mentioned by Garvey (1977). David, a 5-year-old boy, laughed and misnamed parts of his face. For instance, he pointed to his forehead and said, “Here is my mouth” to mock his 2-year-old sister, who had previously shown an adult her likely newly acquired ability to identify parts of her face (i.e., eyes, nose, and mouth). In Garvey’s case, the target is the little sister. In other situations, the targets may be strangers or other family members. Typical cases may involve imitating adults’ funny behavior or appearance, such as a grandfather snoring or a mother putting on makeup.

Consider an example from my *corpus*. A 3.3-year-old boy, exaggerating his mother’s thinking mood, says, “Let us see, let us see…” (Vediamo un po’, vediamo un po’….).

However, older children also use forms of humor typically used by younger children, such as offer/withdrawal: “Mom, I brought you a cookie!” says a 4.7-year-old boy. When the mother, thanking him, approaches her son to obtain the cookie, the child eats it.

The following example illustrates a case of playing with expectations regarding new skills: a 6.3-year-old boy tells his mother, “Today the teacher scolded me because I was not able to read…I got an A! (10 e lode).”

The following example demonstrates play with relational expectations. A mother reports an incident with her 6.5-year-old daughter: “We are at the table, and my daughter looks at us and says, ‘You are old, but dad is the oldest in the house! Ah, ah! I am kidding, you are the most beautiful parents in the world,’ and she gets up and hugs us.”

The following example is of disrupting others’ activity (3.1-year-old girl): the grandmother is counting money aloud, and her granddaughter says numbers at random to confuse her.

In conclusion, teasing represents an intriguing form of humor because it develops precociously, yet older children and adults also use it. Keltner et al. (2001) conducted a comprehensive review of this form of interaction at different ages and proposed the following definition: teasing is intentional provocation accompanied by playful markers that together comment on something relevant to the target.

Consider the following instances of other forms of humorous interactions drawn from parents’ reports.

(1) “The child, looking at the rain, says, ‘What a beautiful day, mom! It is ideal to go to the beach!’ (Che bella giornata mamma! È proprio l’ideale per andare al mare!).” (girl, 6.5 years old)

(2) “I said, ‘Today I have prepared pizza,’ and my child said, ‘Mom, how disgusting! You made the wurstel pizza!’ (Mamma che schifo! Hai fatto la pizza con i wurstel!), his preferred pizza, and he plunged [toward his plate] to eat it (e si è tuffato a mangiarla).” (boy, 5.3 years old)

(3) “Today, during lunch at [the] grandparents’ [home], grandfather made [a] noise when eating his spaghetti. She [the girl] started laughing and said, ‘Grandpa you are very elegant!’ (Lei ha cominciato a ridere e a dire ‘Nonno sei molto elegante!’).” (girl, 5.3 years old)

(4) “She says, ‘Mom let’s go to the beach. Today we have a fine weather’ (Mamma andiamo al mare! È bello oggi il tempo!), while outside it rains like all the other days.” (girl, 4.1 years old)

(5) “Sofia dropped her glass of water, and she looked at me in a loving mood (modo amorevole) and said, ‘It was not me! It was the glass, which did not want to live anymore!’ (non sono stata io! è stato il bicchiere che non voleva più vivere).” (girl, 4.1 years old)

(6) “While I was doing the dishes, two glasses slipped from my hands and broke. Martina immediately commented, ‘Eh, mom, you are really good at doing the dishes!’ (Ehi, mamma, ma come sei brava a lavare i piatti!).” (girl, 3.8 years old)

(7) “He plays with the sand, rolling over (rotolandosi) and getting dirty all over (sporcandosi tutto); he makes castles with a bucket and spade. I tell him, ‘You really like playing with sand, eh?’ And he says: ‘No, I don’t like it at all!’ (No, non mi piace affatto!).” (boy, 3.0 years old)

(8) “Today, while she was playing with her doll, she said, ‘Mom, she is your child! She wants chocolate, she, no me!’ (Mamma lei è tua bimba! Vuole cioccolata… lei, no io!).” (girl, 2.3 years old)

(9) “At lunch, she spilled water all over herself. She started laughing and said, ‘Bath I take!’ (Bagnetto ho fatto!).” (girl, 2.3 years old)

Examples 1–3 appear to be typical ironic utterances. In fact, the children who performed them were in the age range in which, according to the literature, we can expect irony production (Pexman et al., 2009; Recchia et al., 2010). Are the other examples also instances of irony? They appear to resemble the previous examples, but they are uttered by younger, sometimes much younger, children. Example 4 is nearly identical to Example 1, the most classical instance of irony. Example 6 is a strong case of irony. The other examples are less typical, though nonetheless recognizable instances. Consider how we would interpret equivalent utterances if an adult had performed them. For instance, someone is finishing a glass of wine with evident pleasure. In response to the remark “So you like white wine, eh?,” the person answers, “No, I don’t like it at all” (semantically equivalent to Example 7). We would consider this answer ironic. Consider now the following example, analogous to Example 9: someone spills a glass of water on his or her T-shirt and, laughing, says, “I took a shower.” This statement seems to be a textbook case of self-irony. If we consider the former two statements ironic when adults utter them, why should we not do so when similar statements are uttered by young children? Admittedly, with young children, we may doubt that the production of humor was intentional.

In the following sections, I discuss different ways of interpreting children’s ability to use and understand these forms of non-literal communication. First, however, I introduce additional examples of humor that children typically perform from a young age.

(10) “Hello, my little doll” (Ciao bambolotto mio). A 4.11-year-old girl uses the phrase “little doll” to refer to her father imitating her mother, who had greeted her with the words, “Hello, little doll” (bambolotta mia). Note that when addressing the father correctly, the child transitions from the feminine form to the masculine form.

(11) A 3.3-year-old boy tells his mother, “Mind that if you don’t behave I’ll call the traffic policemen” (Guarda che se non ti comporti bene, chiamo i vigili!). In this case, the child employs a typical expression used by Italian parents to calm overly excited or misbehaving children.

Examples 11 and 12 are noteworthy because the humor is produced by the fact that children use expressions with parents that the latter normally use with them and that become incongruous in this inverted form.

Consider another example:

(12) A 6.7-year-old girl is seated with a group of adults and children at a coffee shop. When everyone orders hot chocolate, she exclaims, “Well…I’ll take a beer!”

In this example, irony results from the child’s incongruous appropriation of a behavior that the child cannot yet perform.

Examples 10–12 indicate that a typical way that children use to produce humor is by uttering a statement that is normal when spoken by an adult but becomes incongruous when uttered by a child. Note that this incongruity applies to different forms of humor. In clowning, we may observe gestures rather than words, such as when a child wears her mother’s heels or walks with a grandparent’s cane. It applies also to teasing, as in Example 11, and to irony, as in Examples 10 and 12.

Based on the analysis of the reviewed examples of humor, the following observations are evident:

- If we consider humor a form of communicative interaction, distinguishing different forms of humor by age is not straightforward. At all ages, we observe both simple and more complex forms of humor. Although, we identify two forms of humor typical of young children, namely, clowning and teasing, we have seen that older children use these forms as well. We also note instances of very young children performing what is generally considered the most sophisticated form of humor, irony.
- Children’s humor is multifaceted, but we can nonetheless detect typical manifestations.

Thus far, we have analyzed forms of humor observed in children of different ages. We have not closely examined the current definitions of humor and irony. The next section discusses these definitions from a more theoretical perspective.

## Humor, Irony, and Teasing

This section presents the theoretical assumptions of my work. I propose that the relationship among humor, irony and teasing may be clarified by considering them different forms of a more general communicative ability that appears early in development and is characterized by playing with others’ expectations.

Definitions of humor and irony and their relationship are widely debated in the literature. In cognitive studies, the most accepted definition of humor derives from the work of Shultz (1976) and McGhee (1979), who claim that incongruity with respect to reality is the source of humor. Divergences exist in the literature regarding the type of relation that a subject must entertain with incongruity to perceive humor. McGhee maintains that the subject must be able to *represent* the incongruity, an ability that children acquire at approximately 18 months of age. Shultz considers a necessary condition to be the *resolution* of incongruity, an ability that children acquire at 6 years of age. Other authors, by contrast, consider the *detection* of incongruity to be sufficient (Pien and Rothbart, 1976). This latter stance opens the possibility that infants also display humor. An incongruity becomes the source of humor when it arises within a playful interaction. Recent research has shown that the social emotional context is fundamental to infants’ humor perception (Hoicka and Gattis, 2012; Mireault et al., 2015). In this relational perspective, humor appreciation cannot be evaluated outside the interaction in which it arises. In general, the relational approach admits infants and very young children to its definition of humor. I aim to extend this approach to forms of humor that older children and adults produce, specifically irony.

Humor is difficult to define, and irony is even more difficult to characterize (Gibbs and Colston, 2007). Since Grice’s definition of irony as a violation of the maxim of quality (Grice, 1975, 1978), many authors have attempted to define irony to give an account of forms of irony that do not result from such a violation. Examples include the echo-mention theory (Wilson and Sperber, 1992), the echoic reminder theory (Kreuz and Glucksberg, 1989), the pretense theory (Clark and Gerrig, 1984), the joint pretense theory (Clark, 1996), the allusional pretense theory (Kumon-Nakamura et al., 1995), the relevant inappropriateness theory (Attardo, 2000), the implicit display theory (Utsumi, 2000), and more recent neo-Gricean accounts (Dynel, 2013; Garmendia, 2015). The fact that no theory has definitively prevailed over others can be attributed to the fact that irony is a multifaceted phenomenon. Thus, every definition explains certain aspects of irony, but no definition can explain all aspects. The same claim can be asserted regarding the function of irony. Some authors contend that irony functions to criticize a behavior in a particularly aggressive way (Colston, 1997; Toplak and Katz, 2000). By contrast, the *tinge* hypothesis views irony as a way to lessen criticism (Dews et al., 1995). Both situations are commonly corroborated by empirical reports. Sometimes sarcasm makes a criticism particularly harsh, whereas other times the mitigating aspect of the indirect form prevails and the criticism is alluded to but not explicitly uttered. A recent study has shown that ironic criticism is perceived as simultaneously more mocking and more polite (Boylan and Katz, 2013). Additionally, the relationship between irony and sarcasm is debated. Some authors use these two terms interchangeably, whereas other authors stress the more aggressive nature of sarcasm that aims to hurt the interlocutor (Lee and Katz, 1998). Other problems are posed by the function of ironic compliments, such as in the utterance “Selfish, as always!” directed toward someone who has just acted generously. Thus, it seems that there are various forms of irony and that irony may have different functions.

However, these distinct theories appear to agree that the recognition of irony always requires shared background knowledge. In fact, it is only shared knowledge that allows one to interpret an ironic utterance as such. This requirement is also the cause of misunderstandings because sharedness is necessarily attributed by actors to each other, and it is always possible for an actor to interpret as shared something that is not (Airenti et al., 1993b). Irony is not only based on shared presuppositions; it may also stress them. “Irony is, in this way, a particularly compelling means of reaffirming presuppositions common to both the speaker-author and the audience” (Gibbs and Izett, 2005). These authors claim that empirical research shows that people use irony to “specifically and succinctly comment on the disparity between expectations or beliefs and what is actually happening.”

Another unresolved question concerns the relationship between humor and irony. As in the case of defining irony, different answers have been proposed in the literature. Some authors suggest that humor and irony share basic mechanisms (Giora, 1995), whereas for others, humor is not the final goal of irony but an associated phenomenon (Bryant, 2012). Gibbs et al. (2014) maintain that it is impossible to discern a direct link between irony and humor, even if laughter (or at least, a smile) may often be associated with irony.

I suggest that the relationship between irony and humor may be clarified if, rather than considering only adults, we analyze forms of humor that young children also use, specifically teasing. Linguists assert that teasing and irony must be considered distinct phenomena, even if irony may be used to tease an interlocutor (Dynel, 2014). Some psychologists have highlighted the teasing aspect of irony (Pexman et al., 2005), but the relationship between teasing and irony is more involved. Following the earlier remarks about irony by Gibbs and Izett, irony can be defined in terms of the disparity between reality and expectations, where an expectation is based on shared presuppositions. From this perspective, irony is a phenomenon continuous with teasing. In fact, the two forms of humor differ only in the degree of complexity of the presuppositions, which can be highly basic in teasing, at least in young children’s teasing, but considerably more sophisticated in irony. Compare irony and teasing with respect to humor. If irony does not necessarily provoke laughter, teasing also need not do so. Teasing, moreover, involves a latent aggressive component that makes the teasing not necessarily amusing, at least for one of the interlocutors. This lack of amusement is clear in the case of disrupting others’ activities, but in other forms of teasing, humor may also originate from the disconcertment (or related feelings, such as disappointment, embarrassment, and fear) displayed by the interlocutor. In such cases, laughter may occur, but it is not always the immediate expression.

Defining humor is complicated by the fact that the boundaries separating its different forms are blurred (Norrick, 1993; Attardo, 1994, 2002). However, if we adopt a cognitive perspective and study humor in development, we notice that very young children display basic aspects that evolve with age. Specifically, I hypothesize that young children learn to play humorous communicative games and that the main cognitive and interactional features of these games persist in adult life.

In other words, I propose that humor is a form of communication. Rather than delimiting different categories of humor in linguistic terms, I suggest analyzing the cognitive and interactive components of humor. I argue that different forms of humor depend on the degree of elaboration of different components that define different types of communicative games.

From this perspective, let us consider the relationship between irony and teasing. Angeleri and Airenti (2014) proposed the following componential definition of irony: irony is a non-literal utterance that is based on a common ground shared between interlocutors, focuses on an unexpected incongruity, and includes a teasing component.

We can adopt this perspective more generally and consider that all forms of humor combine different constituents that may co-occur to different degrees. Different communicative games arise from these constituents. Without claiming to be exhaustive, the following examples demonstrate such cognitive-interactional constituents:

- Different degrees of teasing, implying different levels of aggressiveness, may characterize different forms of humor, ranging from mild irony to cruel sarcasm.
- Different games may select different targets of teasing, from the actor herself in self-irony to the interlocutor or a third party.
- Different degrees of indirectness may be possible. Note that the much-discussed example “I love children who keep their rooms clean” is only apparently a literally true utterance. Rather, it is an indirect speech act because the mother is reproaching her child for not having cleaned his or her room.
- Games might differ with respect to the degree of straightforwardness and spontaneity of the communicative acts (with the aim of generating laughter and amusement) and the degree of premeditation (e.g., a sarcastic expression can be carefully planned to hurt the interlocutor).
- Different games may depend on the degree of complexity of knowledge that constitutes the common ground enabling the expectations, which are unfulfilled (e.g., explicit beliefs or implicit background assumptions).

Because all of the identified components are already present in young children’s teasing acts, I propose that teasing is the prototypical form of humor.

Therefore, we can draw the following two conclusions:

- If regarded as communicative games, different forms of humor cannot be differentiated by age.
- All forms of humor, even the more sophisticated instances, can be characterized by playing with expectations, and every form of humor includes a teasing component.

## The Development of Humor in Communication: The Role of Theory of Mind Abilities

In the developmental literature, a clear distinction has been proposed between the acquisition of spontaneous forms of humor, which is typical of infants and young children, and sophisticated forms of humor, including irony. The use of simple humor has been observed in children’s familiar contexts. For these forms, the problem of comprehension has not been posed. By contrast, the comprehension of sophisticated forms of humor is considered a conceptual attainment that must be assessed with classical experimental procedures. Most experimental studies have shown that children’s understanding of irony does not begin before 5 or 6 years of age (Dews and Winner, 1997). According to the few published studies on this topic, production likewise begins at this age (Pexman et al., 2009; Recchia et al., 2010). Only Recchia et al. found examples of hyperbole in 4-year-olds^2^ that could be considered a display of irony. In these studies, observations were completed for a predefined limited time in specific contexts.

The late acquisition of irony is explained in terms of the ToM. The comprehension of irony implies the attribution of second-order beliefs to the speaker, or a full-fledged ToM (Winner and Leekam, 1991; Sullivan et al., 1995; Hancock et al., 2000; Filippova and Astington, 2008, 2010). However, as the previous sections demonstrated, instances of children’s humor in natural situations show that young children also make utterances that would be defined as ironic when performed by adults. Thus, one can argue that these utterances may *seem* ironic, but in claiming that they *are* ironic, we would be attributing to the child an intentionality that has not been proven. Considering these utterances ironic would constitute an over-interpretation. This perspective is supported by the fact that in experimental studies, young children do not seem to understand the ironic character of utterances.

I believe these two assumptions should be questioned. On the one hand, it is not clear that adults produce ironic utterances deliberately (Gibbs, 2012). It has been proven that adults may comprehend the meaning of an ironic utterance without explicitly recognizing its ironic character (Gibbs and O’Brien, 1991). We rather expect that a communicative act be used appropriately. Our data indicate that young children may sometimes use ironic utterances appropriately. On the other hand, recent experimental studies have shown that children as young as 3 years old can understand the communicative, non-literal intent of ironic utterances (Loukusa and Leinonen, 2008; Angeleri and Airenti, 2014).

The previous considerations prompt us to reconsider the relationship between the use of sophisticated forms of humor and ToM abilities. A result of this reconsideration might be to extend the concept of ToM. A number of recent studies have shown that infants can attribute epistemic states to agents, including false beliefs (Onishi and Baillargeon, 2005; Luo, 2011). These findings support the hypothesis that psychological reasoning, or an abstract capacity to represent and reason about false beliefs, emerges early in infancy (Baillargeon et al., 2016). This reasoning capacity, often characterized as *implicit* (i.e., intuitive), would persist in older children and adults when the capacity of explicit reasoning has developed.

These results are abundantly debated in the developmental literature. The core of the debate centers on resolving the discrepancy between these results and the fact that 3-year-old children fail the classical false-belief tasks (Low and Perner, 2012; Perner and Roessler, 2012). More generally, the problem entails explaining the relationship between the capacities exhibited by infants during spontaneous tasks and the capacities that older children and adults display when they are requested to perform verbal ToM tasks. Two questions are fundamental with respect to this problem. One question concerns whether precocious abilities are mentalistic. The second question concerns the role of language acquisition and executive functions in the development of more mature reasoning skills.

To explain the discrepancy between infants’ and older children’s performances on false belief tasks, Butterfill and Apperly (2013) postulate the existence of two distinct systems. Before being able to represent mental states, children would develop a minimal ToM, an efficient yet inflexible system implied in precocious social abilities. The researchers assume that a minimal ToM involves representing belief-like states but does not involve representing propositional attitudes as such. Therefore, due to its limitations, this system would be unable to deal with complex sets of mental states.

San Juan and Astington (2012) note that no plausible theory exists to explain how children progress from implicit (i.e., automatic) reasoning to explicit (i.e., controlled) reasoning. In particular, they stress the possible role of social and linguistic experiences in facilitating this progression.

Other authors have emphasized the influence of social experiences, which may help to explain individual differences in the development of ToM abilities (Apperly, 2012; Hughes and Devine, 2015). Also Baillargeon et al. (2016) concede that we possess insufficient knowledge regarding the development of infants’ ability to infer and reason about others’ mental states and the factors that contribute to individual differences.

Kovács et al. (2010) conducted a series of experiments that supported the hypothesis of a typically human attitude to encode others’ beliefs. They showed that the mere presence of social agents is sufficient to automatically trigger online belief computations in both 7-month-old infants and adults. On the other hand, some studies show that in false belief tests, perspective tracking can be disrupted by the request to use explicit language, a phenomenon also attested in both children and adults (Rubio-Fernández, 2013; Rubio-Fernández and Geurts, 2013). Considered together, these findings support the assumption that intuitive and explicit reasoning are alternative forms of reasoning, even though they may coexist in principle.

I believe these findings show that young children are able to monitor others’ behavior and to adapt to their actions regardless of how precocious forms of intuitive comprehension of agents’ actions are characterized (Airenti, 2015). Studies on intersubjectivity have shown that very precociously young children can interact with adults (Trevarthen, 1998). It is reasonable to believe that this finding implies that young children react to adults’ behavior. However, no evidence exists that infants can represent propositional attitudes as such. Moreover, it is not clear that implicit reasoning can be considered indicative of having developed a minimal ToM. At issue is more than a terminological problem; defining implicit reasoning as evidence of ToM, though a minimal form thereof, hides the specific nature of intuitive reasoning about others, namely, the fact that such reasoning develops in interactions and is inseparable from the communicative intentionality characterizing infant behavior. In naturalistic situations, perspective tracking is one way of establishing common ground that enables communication. Other ways exist, such as emotion recognition, which is particularly relevant for acknowledging a playful interaction and, subsequently, humor.

Hence, I argue that we must postulate not precocious ToM abilities but precocious communicative abilities. Precocious communicative abilities allow young children to interact with others efficiently and to enter what has been called a *community of minds* (Nelson et al., 2003; Nelson, 2005).

Humor is a form of communication that children acquire as they do all other forms of communication. They perform it in their interactions with adults and, later, with peers. Developmental pragmatics assumes that children acquire speech acts, or communicative units, that initially entail only acts and subsequently include language and acts (Bruner, 1975). Two facts are crucial to consider. First, children acquire communicative acts simultaneously with the conditions of their use, that is, communicative formats (Bruner, 1983) or games (Airenti et al., 1993a; Airenti, 2010). Second, they learn that playing with the conditions of applicability of communicative acts can generate amusement. Thus, we can provide an interactional definition of how the unexpected that creates humor is produced, namely, by playing with the conditions of applicability of a communicative game. This definition explains why children may use non-literal communication in interactions yet be unable to define its features.

The observation of infants has shown that humorous interactions occur in the first year after birth (Reddy, 1991). Already at this preverbal age, children play with others’ expectations. The simplest and most common children’s social game is peek-a-boo. With respect to the categories previously mentioned, it is the mildest form of teasing. It provokes immediate laughter and is based on shared knowledge of the immediate physical surroundings.

Other precocious typical teasing games combine acts and language. Adults help familiarize children with these formats. Consider, for instance, the use of nursery rhymes. In many cultures, parents perform nursery rhymes, which prompt the child to *expect* a particular *unexpected* event, namely, tickling. The following is a common English example:

Round and round the garden
like a teddy bear
one step, two step
tickle you under there!

This nursery rhyme associates a simple story with movements performed on the child’s arm that result in a teasing episode, which provokes laughter. In this case, the child learns to play with expectations using both gestures and words in an already elaborated manner, compared with, for instance, the simpler game of peek-a-boo.

What children acquire is a specific form of communication, namely, teasing, which is performed by playing with others’ expectations. Parents are often amazed by the creativity of their children. In fact, if we examine the literature and our *corpus* of collected data, we may be surprised to find a repeated appearance of a limited number of communicative games that have a teasing quality and that most children play. As discussed, children play with others’ feelings and with expectations regarding their own abilities. They mock others by imitating and ridiculing their behavior. They can negate an understanding that is evidently shared. They may justify their own mistaken or clumsy behavior by redefining it in an amusing manner. They provoke laughter by assuming an adult’s stance. All of these communicative games are continuous with forms of humor that have a teasing component and are classified as irony or sarcasm when performed by adults.

According to the existing literature, young children cannot distinguish between non-literal communication and lies. In fact, children’s immature ToM prevents them from explicitly expressing this distinction. However, this fact does not reveal insight regarding their capacity to produce and comprehend non-literal communicative acts (i.e., to use these communicative acts appropriately). It is reasonable to infer that a 3.3-year-old girl who laughs and tells her mother, “I am not sucking my thumb,” while actually sucking her thumb is not lying but playing a teasing game. If the child intended to lie, she would have employed a hiding strategy, regardless of its naiveté or effectiveness. As noted, other typical situations exist in which the use of irony constitutes an alternative to lying—for example, when a child cannot conceal a wrongdoing such as not having eaten a disliked food or having soiled an article of clothing. In these cases, irony is used as a possible escape. As the 3.3-year-old girl who soiled her T-shirt with ice cream says while looking at her mother, “This way, I can also eat the ice cream at home!” (‘Così mangio il gelato anche a casa!’).

I have argued that the results of numerous experimental studies indicate only where young children fail in attempting to interpret non-literal communication. Children are unable to explicitly appreciate the nature of non-literal communication. We can now add what young children can accomplish with respect to this particular form of non-literal communication: humor. Children intentionally produce teasing communicative acts. Among these forms of teasing communicative acts, we also find incipient forms of irony.

Consider a hypothetical example of irony in adult communication. Two colleagues are standing near a coffee machine and see another colleague approaching. One of them exclaims, “Here is the hidden gem!” This exclamation is a perfect example of a “specific and succinct” comment about a situation in which the richness derives from the presuppositions shared between the two interlocutors. We can imagine that the two colleagues share the knowledge that the newcomer is particularly praised by his superiors, that he considers himself worthy of this consideration and that they, on the contrary, do not think that he deserves it. Let us now consider an actual example from our *corpus* that involves a young child. A 3-year-old boy is playing in a park. He sees another little boy approaching and tells his mother, “Look! My friend is coming!” Here, too, is a specific and succinct comment about a situation. The difference from the earlier example is that the presuppositions are simpler: the boy shares with his mother the knowledge that he does not like the other boy.

In conclusion, children may produce and understand non-literal communication without having developed a full-fledged ToM. Teasing communicative games do not differ in principle from serious communicative games; in fact, they are acquired in an identical way and become increasingly complex with age. Many elements may relate to their development, such as an improvement in language abilities, executive functions, social and ToM skills. Importantly, however, the considerably more sophisticated adult forms of communication involve the development of communicative formats that are already present in childhood.

## Discussion

This work aimed to delineate a developmental framework for humor. The intent was twofold: to examine the different forms that humor can assume in childhood and to use the analysis of children’s humor to illuminate typical problems that are debated in research on humor in general.

The existing literature typically distinguishes between spontaneous forms of humor, which infants and young children perform, and refined forms of humor, which only older children and adults can produce and understand. Based on this distinction, young children would be able to perform only forms of humor that generate immediate laughter. Other forms of humor that adults perform, such as irony, are generally regarded as considerably more complex and thus beyond the capacity of children to perform. In particular, young children are regarded as lacking the ability to produce and understand such forms of humor because they cannot comprehend their non-literal character. This task of comprehension would require inferring others’ mental states and, hence, the development of a full-fledged ToM.

If, however, we conduct naturalistic observation, the situation appears differently. When asked to record their children’s humorous interactions in everyday life, parents report that even young children may use complex forms of humor appropriately. I have contended that young children acquire complex forms of humor within communicative games and that this acquisition does not require the ability to explicitly express an understanding of the implied mental states.

I argue that to more fully comprehend the problem of acquiring complex forms of humor, it is helpful to analyze what humor entails as a form of communication in general. To facilitate this analysis, I proposed to focus on a form of humor that begins developing very early in life and is present in a more advanced form in adults, namely, teasing. I discuss several examples of teasing that are typical of different ages. Reddy (1991), who studied teasing in infants, defined teasing as playing with others’ expectations. I propose that playing with others’ expectations by teasing should be considered the crucial feature that characterizes humor in general and constitutes the link between the simplest and the most sophisticated forms of humor. Teasing might thus be considered the prototypical form of humor from which irony and sarcasm also arise. In fact, even if teasing and irony are considered distinct forms of communicative acts, it is widely accepted that irony may include a teasing component.

Thus, I propose the possibility of a basic form of human communication characterized by playing with others’ expectations by teasing. Children acquire this form of communication very early in their interactions with adults. Such communication games may assume different forms that become increasingly sophisticated during development, aided by the development of ToM abilities. However, even young children may acquire communicative games that include sophisticated forms of humor and use them. For instance, irony may be part of a game of justification. Children may know that a parent who laughs at a self-ironic description of a misdeed will likely be more indulgent and will abstain from scolding the child. Another example is the appropriation of a communicative game typical of adults. This is a simple move, and it is clear that this appropriation is unexpected and provokes laughter in the audience. We can thus see that acquiring the ability to perform more complex acts of humor does not differ from the way an interactional perspective explains the acquisition of the ability to perform the simplest acts of humor. Consider the case of a child who discovers that if she wears her mother’s shoes, she will provoke laughter in the audience. These acts exemplify young children’s communicative cleverness. This cleverness is not apparent if we ask children to provide explicit explanations of conceptual differences. The same claim could be made of other communicative acts. We do not doubt that when children make a request, they produce an intentional act, even if they are unable to define a request as a communicative act.

Therefore, we can assert that in early stages of development, a child’s use of the communicative game is deliberate, whereas irony is not (Gibbs, 2012). Later in development, acquiring ToM in connection with linguistic proficiency and other cognitive capacities enables the performance of more elaborate forms of humor and the possibility of using communicative games more flexibly.

From a cognitive perspective, the degree of complexity of the different forms of humor depends on the complexity of the communicative game. Thus, rather than claiming that one form of humor is simple while another form (irony, for instance) is difficult, I suggest that the difficulty or simplicity of producing and comprehending a given instance of humor derives from the combination of several constituents that construct the specific communicative game. Most remarkable among these constituents is common ground. Every utterance draws its communicative meaning from a common ground that the interlocutors share. Common ground constitutes the context for comprehension. Nonetheless, the aspects considered when identifying common ground may differ considerably (Clark, 1996). For instance, the common ground that is merely the immediate physical context (what the interlocutors see or hear, for instance) differs notably from one that is an element of general knowledge. In Angeleri and Airenti (2014), we showed that children more easily understand the communicative intent of ironic utterances when the common ground is directly perceived by the interlocutors (contingent irony) than in situations in which irony is based on background knowledge that the interlocutors are supposed to share but that is not directly perceived or mentioned (background irony).

Another factor that may influence the ease of comprehension is the degree of indirectness. Planning an indirect act to hurt someone’s feelings, as in the case of sarcasm, is considerably more difficult than directly mimicking an interlocutor’s behavior to ridicule him or her.

At least two research directions are apparent. I propose several characteristics as relevant for defining different forms of humor. However, it is possible that other characteristics could be considered. I contend that such additional characteristics would make the present model more elaborate but would not invalidate it. Another direction that could be examined in depth is the relationship between comprehension and production. Are these two processes symmetrical in acts of humor? Only a systematic study could indicate whether production and comprehension develop simultaneously.

Comparing production and comprehension is not easy because of the different methods that may be utilized to study these two aspects. With respect to the production of humor, the only effective method is an observation technique. We cannot provoke the use of humor in an experimental situation. Moreover, we must resort to parent reports, which are observations made by non-professional observers. Naturally, parents are given precise instructions; for instance, they are asked to describe the context in which any specific humorous utterance is produced. The main problem involved in the use of this method is that it does not allow precise quantitative analysis because it is impossible to ensure that all parents devote the same attention to the observation of their children’s behavior. However, these limitations are balanced by the possibility to access the child’s spontaneous behavior at any time. I expect that future work will confirm that even very young children use a wide range of humorous utterances. Moreover, I expect to find similar typologies of humor in all children, namely, the forms that we have observed in our sample.

In contrast, comprehension can be assessed through experiments. Experiments may also be used to evaluate the factors that influence performance in humor tasks. According to the theoretical assumptions expressed in this paper, one would expect no direct correlation between performance in humor tasks and performance in ToM verbal tasks. This is the result that we obtained in Angeleri and Airenti (2014). In this study, we tested children aged 3.0–6.5 years in a task of comprehension of different forms of humor. Children were administered the Peabody Picture Vocabulary Test – Revised (PPVT-R; Dunn and Dunn, 1981; Italian adaptation: Stella et al., 2000) and three classical ToM tasks of first and second order: the Smarties task (Perner et al., 1987), the Sally-Ann task (Wimmer and Perner, 1983), and the ice-cream van story task (Baron-Cohen, 1989). To identify the specific effects of ToM and language on humor comprehension, we used path analysis. Our analyses suggested that the correlation between humor understanding and ToM was spurious, as indicated by the shared effects of language ability on ToM and humor and by the shared indirect effects of children’s age on language and ToM.

My perspective is compatible with the point of view expressed by Reddy (2007) that intentional insincere communication is acquired alongside with intentional sincere communication. My perspective differs in its approach to insincere communicative acts. I suggest that two different forms of insincerity must be distinguished: proper deceit and non-literal communication. From a pragmatic perspective, we may regard non-literal utterances as instances of insincerity because they violate the Gricean maxim of quality. However, these different forms of insincerity are acquired differently. Planning a deceit requires the use of ToM abilities, whereas the other forms of insincerity are precociously developed as part of children’s communicative repertoire.

I believe my perspective is advantageous to explain the fact that young children may produce sophisticated forms of humor, as the empirical evidence shows, without attributing them ToM abilities that are not demonstrated in other domains. The latter is true particularly for deceit, which children do not perform until a later age (Peskin, 1992; Airenti and Angeleri, 2011; Lee, 2013). Acquiring the ability to play communicative games is unrelated to acquiring the ability to distinguish true from false statements and to instill false beliefs in others. As shown, in communicative interactions, young children use non-literal communication, particularly humorous communication, as an alternative to lying.

This perspective is also useful for obtaining a better understanding of humor in general and of the relationship among humor, irony and teasing in particular. Studies on humor aim to define and categorize the different forms of humor. They encounter difficulty with the fact that humor manifests multifariously and that it is difficult to formulate definitions that allow the construction of a categorization without overlaps. The fact of laughter cannot be a criterion because several forms of humor exist in which an association with laughter is indirect and even loose or absent. We also cannot identify a function that characterizes all forms of humor. Sometimes humor represents a simple way to express immediate amusement, whereas other times it may function primarily to strengthen the relationship between the interlocutors by stressing and confirming shared knowledge. It may also be used to indirectly criticize an interlocutor in ways that can range from mild to harsh.

I propose the construction of a unifying cognitive framework underlying the communicative games from which different manifestations of humor arise. I argue that this framework can be constructed by analyzing one of the most precocious and pervasive forms of communication, namely, teasing. Teasing is a feature that can be found in all forms of humor, whether simple or complex. I thus propose to characterize humor as a form of communication that has a teasing component and that plays with expectations. Children acquire this general communicative format during their initial interactions with adults. This communicative format becomes increasingly flexible and articulated with age and with cognitive acquisitions, including language abilities, ToM and relational competence.

Consider a final example of a child’s utterance. When his mother’s car does not start, the 3.6-year-old boy asks, “Are we going to sleep here, mom?” How might we determine, in this case, whether this utterance is ironic? I would propose this remark as a typical form of teasing and, therefore, of humor.

## Author Contributions

The author confirms being the sole contributor of this work and approved it for publication.

## Conflict of Interest Statement

The author declares that the research was conducted in the absence of any commercial or financial relationships that could be construed as a potential conflict of interest.

## Footnotes

1 ^1^Unless otherwise specified, all examples of young children’s humor production presented in this paper are from parents’ reports collected under the supervision of the author in various Italian regions. We instructed parents of children aged 2–6 years to record all humorous communicative acts produced by their children in a given month and the context in which they were produced. We conducted a quantitative analysis on the reports of 90 children (Airenti and Angeleri, submitted). However, the examples presented here are derived from a larger sample of 300 reports. The author thanks the families who participated and Giulia Giacone, Sara Ferrero, Caterina Mancini, and Rachele Barresi for their assistance with collecting and coding the reports.

2 The status of hyperbole is discussed in the literature. Although it has been traditionally associated with metaphor and irony, recent work designates hyperbole as a distinct figure of speech (Carston and Wearing, 2015).
